# Ginger Extract-Loaded Sesame Oil-Based Niosomal Emulgel: Quality by Design to Ameliorate Anti-Inflammatory Activity

**DOI:** 10.3390/gels8110737

**Published:** 2022-11-14

**Authors:** Marwa H. Abdallah, Hanaa A. Elghamry, Nasrin E. Khalifa, Weam M. A. Khojali, El-Sayed Khafagy, Amr S. Abu Lila, Hemat El-Sayed El-Horany, Shaimaa El-Housiny

**Affiliations:** 1 Department of Pharmaceutics, College of Pharmacy, University of Ha’il, Ha’il 81442, Saudi Arabia; 2 Department of Pharmaceutics and Industrial Pharmacy, Faculty of Pharmacy, Zagazig University, Zagazig 44519, Egypt; 3 Department of Pharmaceutics, Faculty of Pharmacy, University of Khartoum, Khartoum 11115, Sudan; 4 Department of Pharmaceutical Chemistry, College of Pharmacy, University of Ha’il, Ha’il 81442, Saudi Arabia; 5 Department of Pharmaceutical Chemistry, Faculty of Pharmacy, Omdurman Islamic University, Omdurman 14415, Sudan; 6 Department of Pharmaceutics, College of Pharmacy, Prince Sattam Bin Abdulaziz University, Al-kharj 11942, Saudi Arabia; 7 Department of Pharmaceutics and Industrial Pharmacy, Faculty of Pharmacy, Suez Canal University, Ismailia 41552, Egypt; 8 Department of Biochemistry, College of Medicine, University of Ha’il, Ha’il 81442, Saudi Arabia; 9 Department of Medical Biochemistry, Faculty of Medicine, Tanta University, Tanta 31511, Egypt; 10 Department of Pharmaceutics and Industrial pharmacy, Faculty of Pharmacy, Modern University for Technology and Information, Cairo 4410240, Egypt

**Keywords:** ginger extract, niosomes, emulgel, sesame oil, quality by design, anti-inflammatory effect

## Abstract

Ginger, a natural plant belonging to the Zingeberaceae family, has been reported to have reasonable anti-inflammatory effects. The current study aimed to examine ginger extract transdermal delivery by generating niosomal vesicles as a promising nano-carrier incorporated into emulgel prepared with sesame oil. Particle size, viscosity, in vitro release, and ex vivo drug penetration experiments were performed on the produced formulations (ginger extract loaded gel, ginger extract loaded emulgel, ginger extract niosomal gel, and ginger extract niosomal emulgel). Carrageenan-induced edema in rat hind paw was employed to estimate the in vivo anti-inflammatory activity. The generated ginger extract formulations showed good viscosity and particle size. The in vitro release of ginger extract from niosomal formulation surpassed other formulations. In addition, the niosomal emulgel formulation showed improved transdermal flux and increased drug permeability through rabbit skin compared to other preparations. Most importantly, carrageenan-induced rat hind paw edema test confirmed the potential anti-inflammatory efficacy of ginger extract niosomal emulgel, compared to other formulations, as manifested by a significant decrease in paw edema with a superior edema inhibition potency. Overall, our findings suggest that incorporating a niosomal formulation within sesame oil-based emulgel might represent a plausible strategy for effective transdermal delivery of anti-inflammatory drugs like ginger extract.

## 1. Introduction

Drug delivery via the transdermal route has enabled delivering a wide variety of drugs for many years [1]. To access the systemic circulation, the drug needs first to travel through skin layers. Afterwards, the bioactive agent is subsequently carried throughout the body via the bloodstream to produce its therapeutic effect. In comparison to the other administration routes, transdermal drug delivery shows potential advantages including avoiding first pass hepatic metabolism, prolonging the duration of action of the drug, reducing fluctuation in drug levels, augmenting pharmacological action, reducing side effects, and improving patient compliance [2]. Most importantly, transdermal drug delivery systems (TDDSs) can be properly used for long-term or chronic use. Consequently, it is a valid choice to develop TDDS for the treatment of a number of pathological illnesses, including inflammation.

Nevertheless, transdermal treatment is only effective with certain kinds of bioactive compounds because of the stratum corneum, which acts as a barrier for the penetration of substances through the skin [3]. As a result, numerous strategies have been implemented to modify stratum corneum permeability. Among them, the implementation of nano-formulations has been acknowledged for the efficacy to bypass transdermal therapy’s limitations [4]. Nano-formulations have been deemed effective TDDSs owing to their inherent characteristics of small particle size, high drug retention, and efficient targeting capabilities. Lipid-based vesicles, such as transfersomes, niosomes, liposomes, and ethosomes, as well as nanoparticles and nano-emulsions, can all be classified as nano-formulations.

Niosomes, also known as non-ionic surfactant-based vesicles, have attracted much attention in pharmaceutical fields as a potential substitute for liposomes as a drug carrier. Despite their comparable features, niosomes offer potential advantages over liposomes including higher stability, lower cost, ease of formulation, and scalability. Furthermore, niosomes have gained popularity as potential transdermal drug delivery systems owing to their ability to alter the stratum corneum’s membrane characteristics, which augment drug penetration through skin layers, and eventually, promote efficient drug delivery to systemic circulation [5]. In topical drug delivery systems, niosomes have been reported to act as penetration enhancers, local drug storage for prolonged drug release, solubility enhancers for drugs with weak solubility, and rate-limiting membrane barriers for controlled delivery systems [6].

Nevertheless, due to the low viscosity of niosomal vesicles, which results in improper skin application, niosomal formulation may provide certain challenges when applied topically [7]. Instead, incorporating niosomes into emulgel preparation may theoretically lead to viscosity increasing of the preparation, encouraging its topical administration [8]. Emulgel is an effective drug delivery vehicle that combines the characteristics of both the emulsion and the gel base [9]. Emulgel possesses the advantages of being easily applied topically and could be intended for transdermal application [9]. Most importantly, emulgel has the capability to improve skin permeability, which in turn, increases therapeutic effectiveness [10]. Therefore, the application of niosomal emulgel is supposed to enhance the penetration of drugs through skin.

Natural products are compounds that can be found naturally or extracted from various forms of medicinal plants. Natural products have historically been used since ancient times as a source of many therapeutic agents. Nowadays, there is a renewed interest for the utilization of natural products for treating a wide variety of disorders, including inflammation. The herbaceous plant *Zingeber officinale*, also known as ginger, a member of the *Zingeberaceae* family, is frequently used as a spice, condiment, and herb [11]. It has been utilized as traditional medicine to treat a variety of diseases, including inflammatory disorders. Gingerols, shagaols, and paradol, the active ingredients in ginger, have been repeatedly reported to exert anti-inflammatory, antioxidant, anti-cancer, and anti-atherosclerotic activities [12].

Additionally, sesame oil, which is made from sesame seeds (Sesamum indicum), and is one of the plant oils, is made of protein, unsaturated and saturated fatty acids, as well as trace amounts of other nutrients such as sesamolin, sesamol, and sesamin in addition to tocopherol [13]. Sesame oil has anti-cancer [14], anti-hypertensive [15], antiaging, antioxidant, anti-inflammatory [16], and immunoregulatory characteristics. Sesame oil can be used to create emulgel [17] and microemulsion [18] by combining it with a variety of carriers. The drug’s delivery and efficacy could be enhanced by combining the sesame oil-based emulgel with a niosomal formulation of ginger extract.

The target of this investigation, therefore, was to formulate ginger extract-loaded sesame oil-based niosomal emulgel. The central composite design was used to optimize the developed niosomal emulgel formulations. Then, the developed formulations were analyzed for the physical properties. In addition, a carrageenan-induced rat hind paw edema test was employed to determine the anti-inflammatory efficacy of the optimized niosomal emulgel formulation.

## 2. Results and Discussion

### 2.1. Characterization of Niosomes Loaded with Ginger Extract

**Figure 1:**
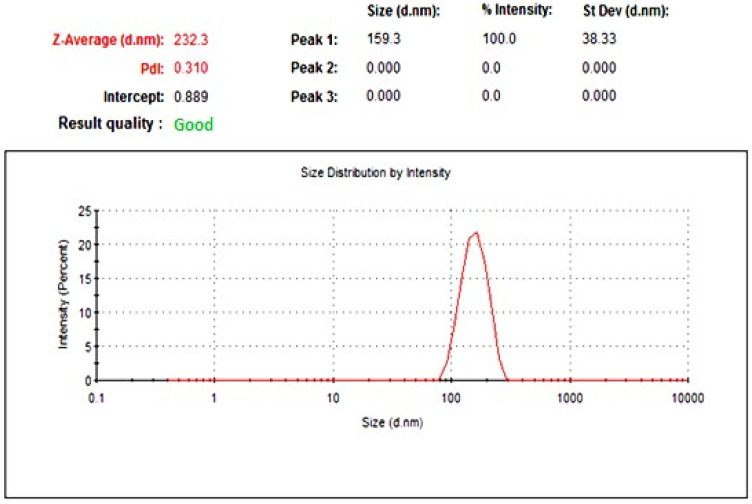
Vesicular size and size distribution curve of ginger extract-loaded niosomes.

Based on our previous investigations, niosomal dispersion with 1:1 molar ratio of cholesterol and nonionic surfactant was developed. Entrapment efficiency of the developed ginger extract loaded niosomes was investigated using centrifugation method. The entrapment efficiency of niosomes loaded with ginger extract was 63.21 ± 2.45%. Particle size and PDI measurement of ginger extract-loaded niosomes were determined, and the results are shown in Figure 1. Ginger extract-loaded niosomes exhibited a particle size of 232.3 ± 3.01 nm, with good size distribution (PDI 0.310).

**Figure 2:**
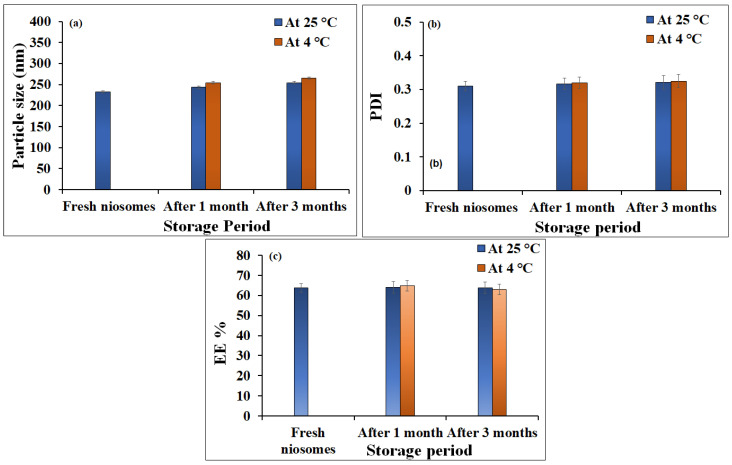
Outline of stability study for ginger extract-loaded niosomal formulation for 1 and 3 months at 4 °C and 25 °C in terms of (**a**) Particle size (nm); (**b**) PDI; (**c**) EE% in comparison to freshly prepared niosomes.

The stability of formulated ginger extract-loaded niosomes, in terms of particle size and entrapment efficiency, was evaluated over one and three months at 25 °C and 4 °C. As depicted in Figure 2, particle size, PDI, and entrapment efficiency (EE) of freshly prepared niosomes did not vary significantly upon storage for one or three months at 25 °C and 4 °C (*p* > 0.05). These results confirmed the stability and capacity of niosomes to transport drugs.

### 2.2. Development of Ginger Extract Niosomal Emulgel

#### 2.2.1. Solubility Studies

**Figure 3:**
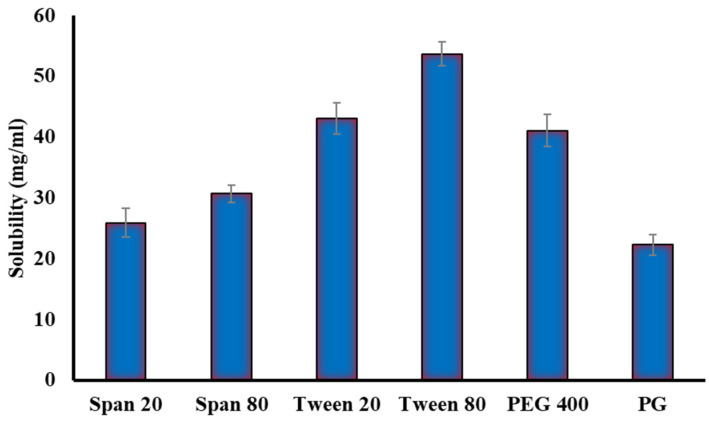
Solubility of ginger extract in different surfactants and co-surfactants.

Solubility investigations of ginger extract in various surfactants (Tween 20, Tween 80, Span 20, and Span 80,) and co-surfactants (Propylene glycol and PEG 400) were conducted in order to select the suitable (S_mix_) surfactant/co-surfactant mixture for the emulsification process. Tween 80 and PEG 400 had the highest solubility of ginger extract when compared to other systems comprising Tween 20, Span 20, Span 80, or Propylene glycol (PG) as illustrated in Figure 3. Tween 80 has the best solubilizing capacity for ginger extract (53.67 mg/mL). Tween 80 is a non-ionic surfactant with high HLB value (14.9) that can be mixed with lipid vehicles to enhance self-emulsification [19]. As a consequence, for subsequent emulsification processes, a surfactant/co-surfactant (S_mix_) comprising Tween 80 and PEG 400 was used.

#### 2.2.2. Construction of Phase Diagrams

**Figure 4:**
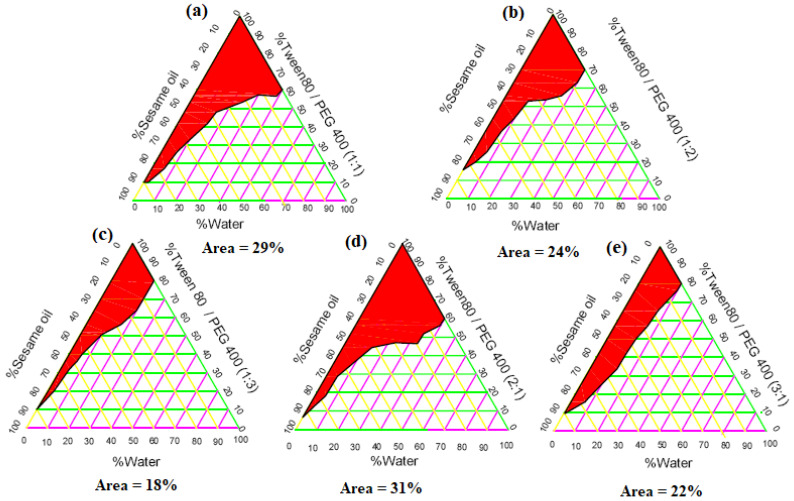
Study of pseudoternary phase diagrams using sesame oil, Tween 80, and PEG 400 at different S_mix_ ratios of (**a**) 1:1, (**b**) 1:2, (**c**) 1:3, (**d**) 2:1, (**e**) 3:1.

The pseudoternary phase diagrams with various volume ratios of Tween 80 to PEG400 (1:1, 1:2, 1:3, 2:1, and 3:1) are depicted in Figure 4. It was evident that the nanoemulsion (ME) area in the phase diagram remarkably increased from 29% to 31% with increasing surfactant to co-surfactant (S_mix_) ratio 1:1 to 2:1 (Figure 4a,d). However, further increase in the S_mix_ to 3:1 resulted in a reduction of the ME area (22%) (Figure 4e). At an S_mix_ ratio of 1:1, the surfactant concentration could not be sufficient to form a closely packed barrier film, whilst a higher surfactant concentration (S_mix_ ratio 3:1) might result in the development of high concentration of micelles, which contributed to the turbidity of the prepared dispersion resulting in the reduction in the ME region [20]. In the same context, at low co-surfactant (PEG400) concentration with respect to Tween 80 (S_mix_ 3:1), the nanoemulsion area was decreased (Figure 4e). The decrease in the nanoemulsion area might be due to the reduced ability of water incorporation to the ME system [19]. On the other hand, increasing co-surfactant concentration in the surfactant/co-surfactant mixture (1:2 and 1:3) (Figure 4b,c) resulted in a remarkable decrease in the ME area (24% and 18%, respectively). The reduced ME areas could be ascribed to low surfactant concentration, which reduced the amount of micelles and decreased the solubilization capacity of ME [19]. Consequently, it was concluded that the maximum ME area was obtained at an S_mix_ ratio of 2:1 and this ratio was chosen for the development of emulgel loaded with the drug.

### 2.3. Central Composite (CC) Design for Emulgel Optimization

Conducting central composite (CC) design software resulted in the generation of 18 runs with four center points. Table 1 clearly shows the impact of different formulation variables on the investigated dependent responses of the prepared niosomal emulgel formulations.

#### 2.3.1. Design Statistical Analysis

Central composite (CC) design was used to conduct the analysis of variance for the investigated dependent responses, and ANOVA was used to acquire specific parameters such as *p*-value, F-value, and model F-value [21]. The quadratic model had the best fit for all responses comparing to all other models. Table 2 shows that most responses had a *p*-value < 0.0001, indicating a significant impact of the independent factors on the tested dependent variables. With regards to the F-value of the responses, it has been proved that higher values are recommended for a model with less error. The F-values for Y_1_, Y_2_, and Y_3_ in the model are 65.72, 122.50, and 31.10, respectively, indicating the significance of the model. Moreover, the lack of fit was 3.73, 5.60, and 1.52, respectively, which were not significant in comparison to the pure error, as well as their corresponding *p*-values, were 0.0943, 0.0935, and 0.3871, for Y_1_, Y_2_, and Y_3_, respectively.

#### 2.3.2. Effect of Independent Variables on Viscosity (Y_1_)

The viscosity of an emulgel formulation is an important criterion since it can change the rate at which the drug diffuses from the vehicle, and thereby could exert a considerable impact on the in vitro drug release [22]. Table 1 summarizes the findings on the viscosity of several prepared ginger extract niosomal emulgels. The viscosity of the formulations fluctuates from 7678.57 ± 137.21 to 10698.20 ± 201.18 cP. It was clear that raising A (gelling agent concentration), B (oil concentration), and C (S_mix_ concentration) resulted in a marked increase in the formulation viscosity, which was most likely related to the formulation compositions [23,24]. The following mathematical equation further demonstrates the noticeable effect of the three independent factors on viscosity:

Y_1_ = −3184.4 + 2205.4A + 31.3B + 1279.65C + 50.2AB − 150.7AC − 8.8BC −156.4A^2^ − 6.1B^2^ − 6.6C^2^ (1)

**Figure 5:**
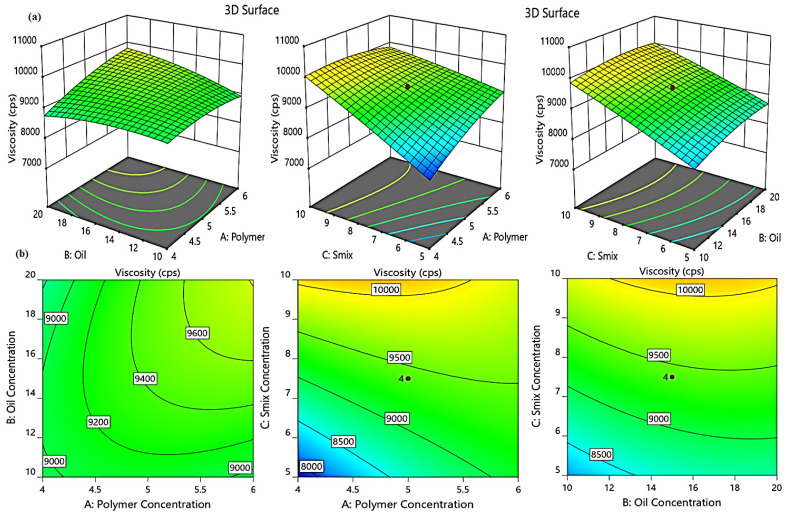
3D response surface plots (**a**) and corresponding contour plots (**b**) showing the effects of the independent variables on viscosity (Y_1_). Two independent variables are considered at a time, while the third one remains constant.

The positive sign in the equation confirmed that A, B, and C had a synergistic effect on Y1 response. In addition, as shown in Figure 5, the positive effect of different formulation variables on formulation viscosity is represented by 3D-response surface plots and 2D contour.

#### 2.3.3. Effect of Independent Factors on the in Vitro Drug Release (Y_2_)

The studies were conducted for 6 h, and the cumulative drug release (%) was calculated. The cumulative drug release ranged from 52.12 ± 0.81% to 80.25 ± 1.92%. It was obvious that the in vitro drug released from all formulations was significantly influenced by the independent variables studied; there was a negative relationship between percent of in vitro drug release and the independent factors. Increasing the polymer (A), oil (B), and S_mix_ (C) concentrations slow down the drug release process from the examined formulations, presumably owing to the higher viscosity which causes resistance to drug dispersion and movement [25]. The following regression equation explains how the independent factors A, B, and C influence the in vitro release response Y_2_:

Y_2_ = 310.72 − 50.62A − 3.32B −24.85C + 0.34AB + 2.08AC − 0.35BC + 2.57A^2^ + 0.14B^2^ + 1.26C^2^ (2)

**Figure 6:**
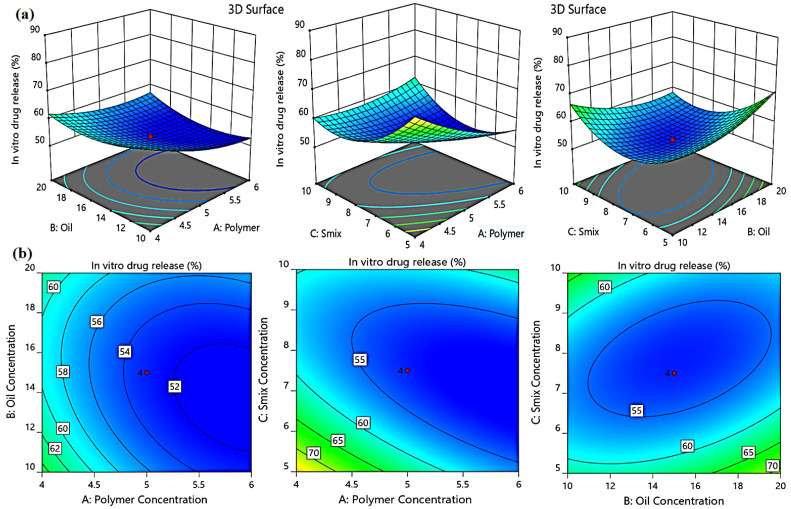
3D response surface plots (**a**) and corresponding contour plots (**b**) showing the effects of the independent variables on the percent of in vitro drug release (Y_2_). Two independent variables are considered at a time, while the third one remains constant.

The impact of independent variables on in vitro drug release (Y_2_) was further studied by the model graphs, as depicted in Figure 6.

#### 2.3.4. Effect of Independent Variables on the % of Drug Content (Y_3_)

**Figure 7:**
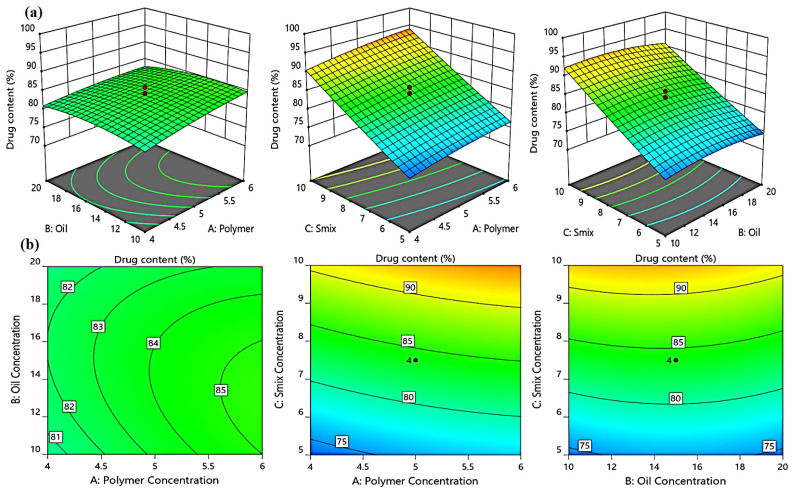
3D response surface plots (**a**) and corresponding contour plots (**b**) showing the effects of the independent variables on the percent of drug content (Y_3_). Two independent variables are considered at a time, while the third one remains constant.

The drug content in the developed emulgel formulations ranged from 70.96 ± 0.46% to 97.58 ± 2.88%, indicating the efficient drug loading within the emulgel formulation. The F-values for drug content (Y_3_) in the model were 1.52 and the *p*-values of the model were less than 0.05, indicating the significance of the model. Equation (3), when expressed in terms of actual factors, can be used to predict the response using different levels of each independent variable. The three independent variables, polymer, oil, and S_mix_ concentrations, had a positive impact on the % drug content, as illustrated in Figure 7.

Y_3_ = 20.67 + 7.63A + 2.24B + 2.89C − 0.13AB + 0.61AC − 0.03BC − 0.45A^2^ − 0.05B^2^ + 0.04C^2^ (3)

#### 2.3.5. Optimized Formulation of Niosomal Emulgel

The analysis of the polynomial model generated by the CC design was utilized to optimize the emulgel development process with fewer formulations. The numerical optimization was applied by the Design Expert^®^ software (version 12.0, Stat-Ease, Minneapolis, MN, USA). The optimized formulation was obtained by orienting the results towards specific characters that expected to accomplish the desired preparation using point prediction technique. The recommended concentrations of the independent variables acquired by the design of experiment to reach to the desired criteria were detected depending on the maximum desirability (0.822). The optimized formula was obtained at a polymer concentration of 4.75%, oil concentration of 10.07%, and S_mix_ concentration of 9.90%. The actual viscosity, in vitro drug release, and drug content were 9510.33 ± 162.79 cP, 64.84 ± 2.35%, and 93.15 ±1.73%, respectively, for the optimized formulation, which were closed to the predicted values (9915.71 ± 136.34 cP, 66.04 ± 1.18%, 91.18 ± 1.95%, respectively).

### 2.4. Characterization of Optimized Niosomal Emulgel Formulation

The parameters of the optimized ginger extract niosomal emulgel were investigated and compared to those of the conventional ginger extract niosomal gel, as shown in Table 3. All the formulations had an appealing appearance with a smooth texture. The pH was in the satisfactory range to avoid possible sensitivity and irritation. In addition, the viscosity of the formulations was investigated, and it was revealed that incorporating sesame oil into niosomal emulgel resulted in significantly greater viscosity than niosomal gel (*p* = 0.006); nonetheless, the viscosity of both preparations was within a good range, supporting topical application [26]. The spreadability of the formulations was tested, and the results proved that they could spread uniformly all over the skin.

Values are stated as mean ± (SD). * *p* < 0.05 compared to Ginger niosomal gel.

### 2.5. In Vitro Release Investigations

**Figure 8:**
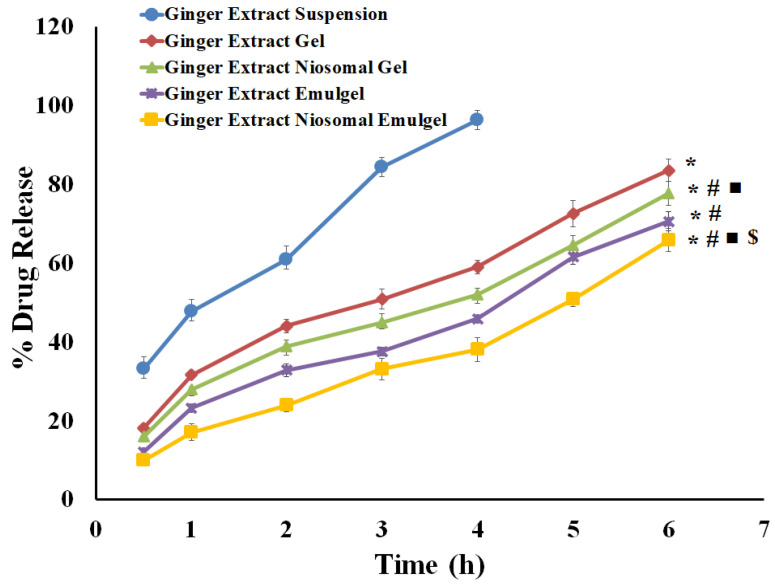
In vitro release study of ginger extract from different formulations compared to ginger extract suspension in phosphate buffer pH 7.4 at 37 °C. Results are expressed as the mean ± SD of three experiments. * *p* < 0.05 compared to ginger extract suspension; # *p* < 0.05 compared to ginger extract gel; ■ *p* < 0.05 compared to ginger extract emulgel and $ *p* < 0.05 compared to ginger extract niosomal gel.

The percentage of ginger extract released from ginger extract suspension and different generated preparations is illustrated in Figure 8. As shown in Figure 8, 96.20 ± 2.44% of the ginger extract in suspension was released within 4 h. On the other hand, a much slower release of ginger extract from various gel formulations was observed. The cumulative release percentage of ginger extract at 6 h from niosomal emulgel, drug emulgel, niosomal gel, and drug gel was 64.84 ± 2.35%, 70.52 ± 2.41%, 77.59 ± 3.02%, and 83.46 ± 2.86%, respectively. The significantly lower cumulative ginger extract release from different gel formulations, compared to free drug, might be accounted for by the existence of a gelling agent, which enhances the thickness or viscosity of the formulation, and thereby restrains drug release from gel formulations. In addition, the amount of Ginger extract released from the gel was substantially greater than that released from emulgel (*p* = 0.004), presumably owing to the higher aqueous content of gel formulations, which facilitates the migration of the drug into the release media. On the other hand, entrapment of the ginger extract within niosomal formulations was found to slow down drug release from niosomal emulgel formulation, compared to Ginger extract-loaded emulgel. The ability of cholesterol in the niosomal formulations to generate cement layer in the voids of the bilayers, resulting in more intact bilayers, could influence the drug diffusion, and thereby slow down drug release [27,28].

### 2.6. Skin Permeation Study of Ginger Extract from Different Formulations

**Figure 9:**
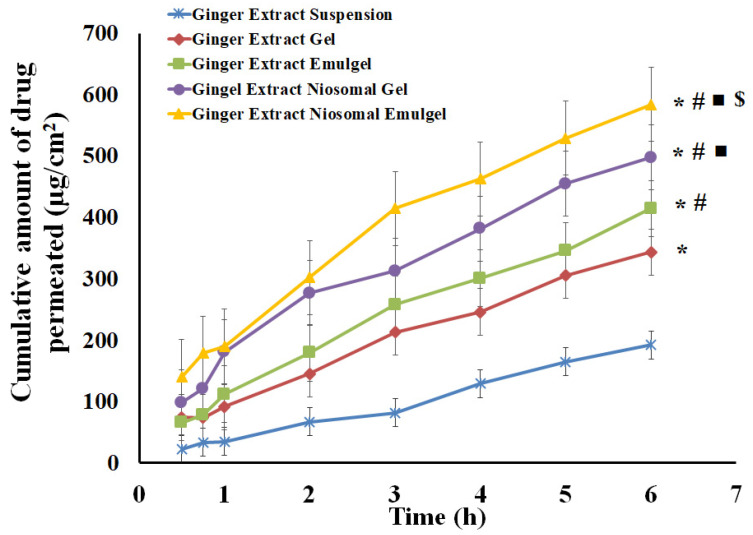
Permeation study of ginger extract from different formulations through excised rabbit skin compared to ginger extract suspension (control). Results are expressed as mean ± SD (*n* = 3). * *p* < 0.05 compared to ginger extract suspension; # *p* < 0.05 compared to ginger extract gel; ■ *p* < 0.05 compared to ginger extract emulgel and $ *p* < 0.05 compared to ginger extract niosomal gel.

The in vitro investigation of ginger extract permeability across excised rabbit skin from the generated formulations was performed and compared to ginger extract suspension. As shown in Figure 9, the highest transdermal permeability was observed with ginger extract niosomal emulgel; the J_ss_ value was (81.84 µg/cm^2^.h), while the lowest transdermal permeability was observed with ginger extract suspension (J_ss_ 30.95 µg/cm^2^.h). In addition, the ex vivo permeation of ginger extract from ginger extract-loaded emulgel was higher than that from gel formulation. The J_ss_ values of ginger extract-loaded gel and ginger extract-loaded emulgel were 62.59 µg/cm^2^.h and 51.24 µg/cm^2^.h, respectively. The higher enhancement ratio of ginger extract from the emulgel formulation, compared to the gel formulation, might be ascribed to the existence of oil and S_mix_, which could act as penetration enhancers [29,30]. Interestingly, the entrapment of ginger extract within niosomal vesicles was found to enhance drug permeability from niosomal gel and emulgel, with ERs of 2.30 and 2.64, respectively. This result could be ascribed to the ability of niosomes to modify the stratum corneum structure and make it looser and more permeable [31]. Furthermore, compared to niosomal gel formulation, incorporating niosomes within emulgel formulation greatly enhances the drug flow as a result of the dual action of surfactant in niosomes and nanoemulsion in emulgel, which further enhances the drug penetration when niosomal preparation is included in the emulgel formulation [32]. Therefore, the aforementioned argument could be used to explain why ginger extract niosomal emulgel is more permeable.

### 2.7. Anti-Inflammatory Testing; Carrageenan-Induced Rat Paw Edema Test

**Figure 10:**
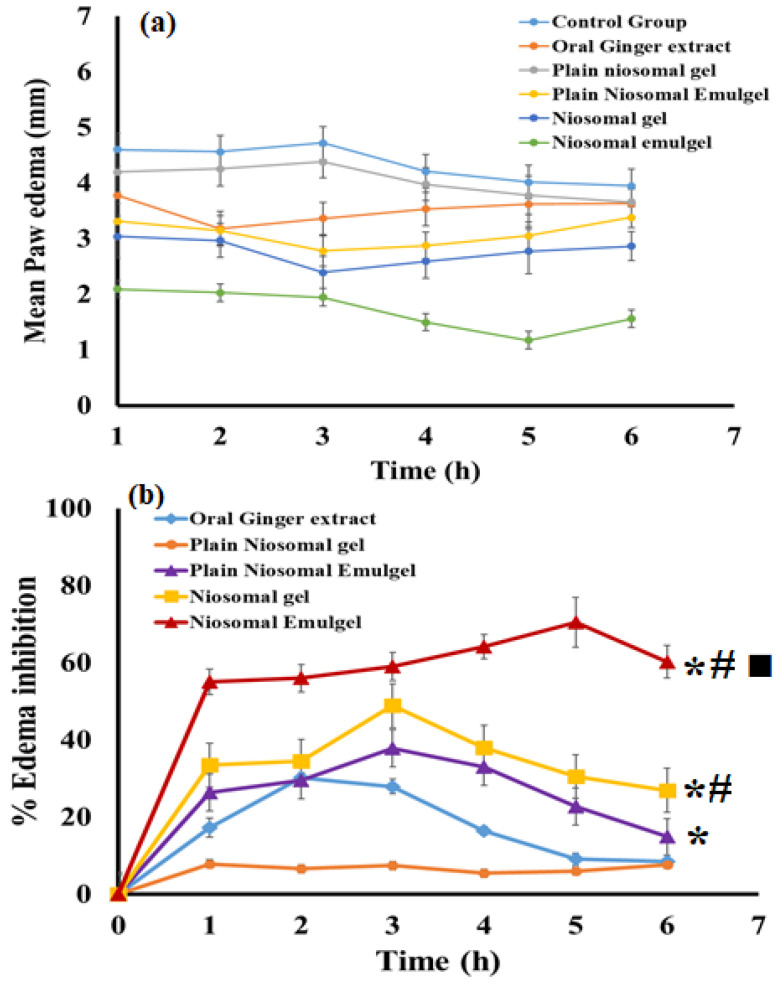
Effects of ginger extract prepared in various formulations on mean hind paw edema (**a**) and percentage of edema inhibition of carrageenan-induced paw edema rats (**b**). Results are expressed as mean with the bar showing SD (*n* = 5). * *p* < 0.05 compared to plain niosomal gel; # *p* < 0.05 compared to ginger extract orally and ■ *p* < 0.05 compared to ginger extract niosomal gel.

Figure 10 displays the changes of size of edema and the percent of rat hind paw edema inhibition induced by carrageenan injection compared to inflamed animals (non-treated control group). The edema size reached its peak within three hours post-injection of carrageenan in the inflamed groups. Similarly, the animals receiving plain niosomal gel formulation (placebo I) reached the greatest inflammation following three hours of starting the test and did not differ significantly if compared to control animals (*p* < 0.05). On the other hand, rats handled with either plain niosomal emulgel (placebo II), ginger extract orally, ginger extract niosomal gel, or ginger extract niosomal emulgel demonstrated a significant reduction in paw edema thickness (Figure 10b) when compared to control animals (*p* < 0.05), where they demonstrated 14.88 ± 1.27% (plain niosomal emulgel), 8.49 ± 1.61% (ginger extract orally), 26.96 ± 2.62% (ginger extract niosomal gel), and 60.27 ± 4.19% (ginger extract niosomal emulgel) edema inhibition after six hours. The difference between the groups treated with ginger extract niosomal gel and emulgel was found to be statistically significant (*p* < 0.05), demonstrating the importance of sesame oil and emulgel in enhancing anti-inflammatory effects. The most notable reduction in edema size was noticed in animals treated with ginger extract niosomal emulgel, which was statistically significant (*p* < 0.05) when compared to the groups treated with other administered formulations. Therefore, the ginger extract niosomal emulgel has the highest potential for anti-inflammatory activity. The current study provides significant insights for the enhanced effect and improved permeability of topical formulations using sesame oil and ginger extract in niosomal emulgel and suggesting a synergistic interaction between them. In a previous study, the potential of sesame oil to reduce inflammation was demonstrated [33].

## 3. Conclusions

In the current investigation, ginger extract-loaded niosomal formulations were created, utilizing the thin film hydration approach. The ginger extract emulgels were produced by combining the formulation with sesame oil-based emulgel using the quality by design methodology. The formulation demonstrated satisfactory physical characteristics, enhanced skin permeability performance, and had considerable anti-inflammatory effects. The results demonstrated the remarkable impact of sesame oil on the in vivo behavior of niosomal formulation, suggesting a synergistic interaction between sesame oil and ginger extract. Finally, niosomal emulgel might be taken into consideration as a potential nanocarrier that effectively delivers the bioactive agents topically.

## 4. Materials and Methods

### 4.1. Materials

Tween 20, Tween 80, Span 20, Span 60, Span 80, polyethylene glycol (PEG) 400, propylene glycol, cholesterol, hydroxypropyl methylcellulose (HPMC), chloroform, ethanol, methanol, and sodium azide were procured from Sigma Chemical Co. (St. Louis, MO, USA). Solvents and chemicals of analytical grade were purchased from Sigma, USA. Sesame oil was provided by NOW^®^ Essential Oils (NOW Foods, Bloomingdale, IL, USA).

### 4.2. Preparation of Ginger Extract

Ginger rhizome, obtained from a local market, was washed with clean water, sliced, and dried at 45 °C for 48 h in an oven and then a fine powder was made. Ginger extract was produced by using a maceration process. Briefly, 100 g of the dried powdered ginger was extracted by 500 mL of organic solvent (methanol), then stirred at 500 rpm by using a magnetic stirrer at room temperature for 24 h, then left to stand overnight at room temperature. Solution was filtered through muslin cloth and then re-filtered through Whatman No.1. The solvent was then evaporated at ambient temperature to produce ginger extract [34]. A full UV spectrum graph for the ginger extract is represented in Figure S1.

### 4.3. Preparation of Ginger Extract Loaded Niosomal Vesicles

The niosomal vesicle formulation was developed using a thin film hydration technique, as described previously [35]. Briefly, in a 50 mL round bottom flask, 100 mg ginger extract was mixed with non-ionic surfactant (Span 60) and cholesterol in (1:1) molar ratio, and then dissolved in 10 mL chloroform/ethanol mixture (2:1 *v*/*v*). Using a rotary evaporator (BÜCHI Labortechnik, Meierseggstrasse, Flawil, Switzerland), the organic solvents were then evaporated at 60 °C under reduced pressure. The dried thin lipid film developed on the wall of the round flask was hydrated with 10 mL phosphate buffer (pH 7.4), then the flask was agitated for an additional hour at 60 °C to obtain niosomal dispersion. 

### 4.4. Characterization of the Developed Niosomal Vesicle Loaded with Ginger Extract

#### 4.4.1. Entrapment Efficiency Determination

The centrifugation method was used to determine the entrapment efficiency of the developed ginger extract-loaded niosomes. In brief, the niosomal dispersion was centrifuged at 6000 rpm for 1 h at 4 °C using a cooling centrifuge [36]. The concentration of the un-entrapped drug in the supernatant was determined spectrophotometrically at λ_max_ of 273 nm [37]. Drug entrapment efficiency percentage was computed using the following equation [35]:

(4) $$\mathrm{Drug}\;\mathrm{entrapment}\;\mathrm{efficiency}\;(\%)=\left[\left(\mathrm{AT}-\;\mathrm{AU}\right)/\mathrm{AT}\right]\times 100$$

where AT is the total amount of dug added and AU is the amount of the un-entrapped drug.

#### 4.4.2. Vesicular Size and Size Distribution

Dynamic light scattering technique was used to assess the vesicular size and polydispersity (PDI) of ginger extract-loaded niosomal vesicles using Malvern Zetasizer (Worcestershire, UK) [38].

### 4.5. Stability Studies

The stability of ginger extract-loaded niosomes was investigated in accordance with the ICH guiding principles. The investigation was conducted at two distinct temperatures: 25 ± 1 °C and 4 ± 1 °C, with humidity of 60% [7]. The efficiency of drug entrapment and size and PDI of niosomal vesicles were measured after one and three months.

### 4.6. Development of Ginger Extract Niosomal Emulgel

#### 4.6.1. Solubility Studies

The saturated solubility of ginger extract in different surfactants (Tween 20, Tween 80, Span 20, and Span 80) and co-surfactants (Propylene glycol and PEG 400) was studied in order to identify solvents with good solubilizing capacity. Extra amounts of ginger extract were mixed in capped 10 mL vials with 2 mL of each vehicle, using a vortex mixer (Fisher Scientific, Waltham, MA, USA), and agitated for 72 h in a thermostatically controlled shaking water bath (Julbo, SW23, Seelbach, Germany) at 25 ± 1 °C. After that, the mixtures were centrifuged at 25 °C at 5000 rpm for fifteen minutes to separate the undissolved ginger extract. Finally, the drug concentration (mg/mL) in the supernatant was detected spectrophotometrically at a maximum wavelength of 273 nm using methanol for dilution. For further investigations, components with the maximum solubility of ginger extract were employed.

#### 4.6.2. Construction of Phase Diagrams

The concentration ranges of the components that potentially result in an extended nanoemulsion area were determined using pseudoternary phase plots. The construction of pseudoternary phase diagrams was employed using a water titration method. Surfactant/co-surfactant mixture (S_mix_) composed of Tween 80 (surfactant) and PEG 400 (co-surfactants) were blended in various volume ratios (1:1, 1:2, 1:3, 2:1, and 3:1). Then, the S_mix_ mixture was blended with sesame oil in various volume ratios (1: 9 to 9:1), using a vortex mixer until the formation of homogenous mixture. Distilled water was added dropwise to the various mixtures until a turbidity was observed. Autodesk^®^ Autocad^®^ program was used to cosruct pseudoternary plots. S_mix_ ratio showed maximum nanoemulsion area was chosen for further studies.

#### 4.6.3. Experimental Modelling Using Central Composite Design

Central composite (CC) design as a tool of response surface methodology (RSM) were implemented for the purpose of formulation optimization. The impact of independent factors on the observed dependent responses was evaluated using Design-Expert software version 12.0 (Stat-Ease, Minneapolis, MN, USA) [39]. As shown in Table 4, a design of three factors with two levels (2^3^) was created utilizing three independent factors depicting concentration of polymer (A), concentration of oil (B) and S_mix_ concentration (C), with 2 levels comprising high (+1) and low (−1). Viscosity, cP (Y_1_), in vitro drug release percent (Y_2_), and percent of drug content (Y_3_) were the dependent responses investigated. To acquire the optimum formula with the intended outputs, eighteen experiments were performed. The analysis of variance (ANOVA) test was used to evaluate the data in order to determine the model’s significance and to demonstrate the data statistical analysis. The influence of the independent factors on the explored dependent responses was analyzed using ANOVA test, which was followed by the creation of model graphs in the form of 3D surface and 2D contour plots, as well as precise mathematical equations. 

#### 4.6.4. Preparation of Niosomal Sesame Oil Based Emulgel

Eighteen formulations were made with different concentrations of gel-forming polymer (hydroxypropyl methylcellulose (HPMC); 4–6%), sesame oil (10–20%), and surfactant/co-surfactant mixture (Tween 80/PEG 400; S_mix_ (2:1); 5–10% *v*/*v*). In brief, different amounts of gel-forming polymer (HPMC) were immersed in 10 mL distilled water and agitated until a uniform hydrogel was developed. On the other hand, nanoemulsion loaded with ginger extract was developed by dissolving the required amount of ginger extract in the required amount of sesame oil with stirring. Then the obtained mixture was mixed with the calculated amount of S_mix_ (Tween 80/PEG 400) for 5 min. Following that, by using a vortex mixer, the aqueous mixture was dripped over the oily mixture with continuous vortexing for 10 min until a nanoemulsion was achieved. The formulated nanoemulsion was mixed with the pre-formulated hydrogel and stirred with the help of a homogenizer mixer (Ika-eurostar 20 high speed digital, Germany) until a consistent sesame oil-based emulgel was generated [40]. The niosomal dispersion was blended with the developed emulgel for achieving the niosomal emulgel encapsulating ginger extract. A niosomal gel containing ginger extract was created to validate the influence of nanoemulsion and sesame oil in our study. A certain amount of HPMC was mixed with ten milliliters distilled water, then agitated thoroughly until a gel was formed, which was then blended with the niosomes to produce a niosomal gel loaded with ginger extract.

### 4.7. Characterization of the Investigated Ginger Loaded Niosomal Emulgel

#### 4.7.1. Visual Inspection

The homogeneity of the generated formulations loaded with ginger extract was visually assessed [41].

#### 4.7.2. pH Value Estimation

At room temperature, a calibrated digital pH meter (PCT-407 Portable pH Meter, Taipei City, Taiwan) was used to monitor the pH of the tested formulation [42]. The pH reading was taken three times and the average was recorded.

#### 4.7.3. Spreadability Test

The goal of this study was to assess the tested formulation’s spreadability by measuring the spreading diameters when they were introduced to the affected area. A specified weight of the tested formulations (1 g) was kept between two glass slides. A definite load of about 0.5 g was applied over the upper slide for one minute. The diameter of the spreading circle was recorded and calibrated in centimeters to determine the spreadability [7].

#### 4.7.4. Viscosity Measurement

The viscosity of the tested formulations was measured at 25 °C using Brookfield viscometer (Brookfield viscometer, Model DV-II, Middleboro, MA, USA). The viscosity measurement in (cP) was carried out three times and the average reading was recorded [42].

#### 4.7.5. Determination of Drug Content

Specified weight of about one gram of the produced test formulations (equal to 50 mg of ginger extract) was accurately diluted to 100 mL using phosphate buffer saline (pH 7.4): methanol mixture (8:2). The drug content was then quantified spectrophotometrically, evaluated at λ_max_ 273 nm [37]. The following formula was used to calculate the percentage of drug content:

(5) $$\%\;\mathrm{Drug}\;\mathrm{content}=\frac{\mathrm{Actual}\;\mathrm{amount}\;\mathrm{of}\;\mathrm{the}\;\mathrm{drug}\;\mathrm{in}\;\mathrm{the}\;\mathrm{formuation}}{\mathrm{Theoretical}\;\mathrm{amount}\;\mathrm{of}\;\mathrm{the}\;\mathrm{drug}\;\mathrm{in}\;\mathrm{the}\;\mathrm{foemulation}}\times 100$$

### 4.8. In Vitro Drug Release Studies

The percentage of the drug released from different manufactured formulations (Ginger extract suspension, ginger extract emulgel, niosomal gel, and niosomal emulgel loaded with ginger extract) was assessed. The in vitro drug release was conducted, using a locally fabricated diffusion cell, as previously described [43] with minor modifications. Cellophane membranes with Molcular Weight cut-off 12,000–14,000 Da, were presoaked in the release medium overnight before use. Accurately weighed amounts (1 g) of each formulation, equivalent to 50 mg of ginger extract, were placed over the dialysis membrane (donor compartment) that covered the diffusion cell and fixed with a rubber band. The diffusion cells were then submerged in PBS with a pH of 7.4, kept at 37 ± 1 °C, and rotated at 50 rpm. Two mL samples were taken at specified periods (0.25, 0.5, 1, 2, 4, and 6 h) and evaluated spectroscopically at λ_max_ 273 nm. To maintain sink conditions, the samples were replaced with fresh buffer of the same volume. 

### 4.9. Ex Vivo Drug Permeation Study

The abdominal skins of white albino male rabbits with thicknesses of 1.1–1.2 mm were employed. Animal skin was carefully removed and processed for the experiment. The full thickness skin was mounted to one end of a glass tube (2.8 cm diameter and 6.15 cm^2^ surface area) with the stratum corneum side facing upwards, while the dermis facing the receptor media. One gram of the investigated formulation containing 50 milligrams of ginger extract were then applied to the donor compartment. Next, the glass tubes were dipped in 500 mL PBS, pH 7.4, containing sodium azide (0.02%), kept at 37 ± 1 °C, and rotated at 50 rpm. At specified time periods throughout 6 h, two milliliter samples were collected and examined at λ_max_ 273 nm using a spectrophotometer. Fresh buffer was added in the same volume in place of the samples. The following equations were used to estimate the steady state transdermal flux (*J_ss_*) and enhancement ratio (*ER*):

(6) $$J_{ss}=\frac{Amount\;of\;permeated\;drug}{area\;of\;permeation\times time}$$

(7) $$ER=\frac{J_{ss\;\left(test\right)}}{J_{ss\;\left(control\right)}}$$

### 4.10. In Vivo Experimental Studies

#### 4.10.1. Animals

Male healthy Wistar rats (220–250 g) were maintained under standardized conditions of temperature, relative humidity, and lighting environment. All experiments were approved by the Research Ethics Committee (REC) at University of Ha’il, Saudi Arabia (Approval no. H-2022-291 at 13/06/2022).

#### 4.10.2. Anti-Inflammatory Activity

A carrageenan-induced rat hind paw edema test was adopted to scrutinize the anti-inflammatory potential of ginger extract incorporated into various formulations. In this assay, paw edema was induced in all rats by subcutaneous injection of 0.1 mL of carrageenan (1% *w*/*v*) into the sub-plantar surface of rat right paw. Rats were then randomly categorized into six groups (*n* = 5) as follows: Group I: non treated positive control rats; Group II: rats orally fed with ginger extract (100 mg/kg); Group III: rats treated topically with plain niosomal gel; Group IV: rats treated topically with plain niosomal emulgel; Group V: rats treated topically with ginger extract niosomal gel; Group VI: rats treated topically with ginger extract niosomal emulgel. The anti-inflammatory potential of different formulations was assessed in terms of paw edema thickness, estimated by subtracting the initial paw thickness at zero time before carrageenan injection from the paw thickness measured at each hour after carrageenan injection [44] using a digital caliper (Quick Mini Gage 0.5, Mitutoyo Cooperation, Kawasaki, Japan). In addition, percentage edema inhibition was calculated as an index of acute anti-inflammatory effect using the following equation: _°_

(8) $$\%\;\mathrm{edema}\;\mathrm{inhibition}=\frac{V_{t}-V_{0}}{V_{t}}\times 100$$

where V_t_ is the thickness of paw edema at time (t), and V_0_ is the thickness of paw edema at zero time.

### 4.11. Statistical Analysis

All values were expressed as mean ± S.D. The differences were compared using one way analysis of variable (ANOVA). *p*-values (<0.05) were considered statistically significant.

## Figures and Tables

**Table 1:** The independent variables used for optimizing different formulations and the detected results of dependent variables.

Formulation	Independent Variables			Dependent Variables		
	A	B	C	Y_1_ (cP)	Y_2_ (%)	Y_3_ (%)
F1	5	15	7.5	9301.23 ± 114.31	53.45 ± 0.69	82.80 ± 1.15
F2	4	10	10	9899.34 ± 127.23	68.86 ± 1.09	91.37 ± 0.24
F3	6	10	5	8388.45 ± 175.22	53.43 ± 1.79	75.62 ± 0.88
F4	5	15	7.5	9378.47 ± 114.10	52.12 ± 0.81	82.27 ± 0.63
F5	6	20	10	9845.65 ± 234.52	62.76 ± 0.74	92.03 ± 1.36
F6	4	10	5	7685.87 ± 128.51	76.40 ± 1.87	70.96 ± 0.46
F7	6	10	10	9287.12 ± 165.32	69.75 ± 1.65	93.81 ± 0.90
F8	5	15	7.5	9465.47 ± 188.24	52.91 ± 1.17	84.38 ± 0.62
F9	5	15	7.5	9345.65 ± 129.65	53.34 ± 0.75	86.04 ± 0.38
F10	4	20	5	7678.57 ± 137.21	80.25 ± 1.92	73.18 ± 0.53
F11	6	20	5	9578.33 ± 145.45	67.28 ± 1.67	72.41 ± 2.32
F12	5	23.41	7.5	9198.10 ± 152.32	64.15 ± 0.83	81.06 ± 1.40
F13	5	15	3.29	7885.34 ± 134.01	78.25 ± 0.97	72.25 ± 1.53
F14	6.68	15	7.5	9387.38 ± 117.08	52.14 ± 0.92	86.82 ± 0.57
F15	5	6.59	7.5	8752.28 ± 115.32	61.82 ± 1.11	80.50 ± 0.73
F16	4	20	10	9645.87 ± 128.24	58.07 ± 1.10	89.37 ± 1.00
F17	3.32	15	7.5	8545.06 ± 136.36	68.05 ± 1.22	78.95 ± 1.53
F18	5	15	11.70	10,698.20 ± 201.18	72.07 ± 1.05	97.58 ± 2.88

A: Polymer concentration (% *w*/*w*); B: oil concentration (% *v*/*v*); C: S_mix_ concentration (% *v*/*v*); Y_1_: viscosity (cP); Y_2_: in vitro drug release (%); Y_3_: drug content (%).

**Table 2:** Statistical and regression analysis results for all responses.

Source	Y_1_		Y_2_		Y_3_	
	F-Value	*p*-Value	F-Value	*p*-Value	F-Value	*p*-Value
Model	65.72	<0.0001 *	122.50	<0.0001 *	31.10	< 0.0001 *
A	51.23	<0.0001 *	171.32	<0.0001 *	9.49	0.0151 *
B	19.72	0.0014 *	0.7694	0.4060	0.2827	0.6094
C	400.03	<0.0001 *	42.02	0.0002 *	263.00	< 0.0001 *
AB	27.15	0.0006 *	17.03	0.0033 *	0.8908	0.3729
AC	61.12	<0.0001 *	154.35	<0.0001 *	0.0488	0.8307
BC	5.18	0.1026	112.76	<0.0001 *	0.2520	0.6292
A²	16.65	0.0040 *	59.78	<0.0001 *	0.6749	0.4351
B²	15.98	0.0040 *	117.11	<0.0001 *	4.75	0.0610
C^2^	1.16	0.2681	565.32	<0.000 *1	0.2354	0.6406
Lack of Fit	5.56	0.0943	5.60	0.0935	1.52	0.3871
R^2^ analysis						
R²	0.9867		0.9928		0.9722	
Adjusted R²	0.9716		0.9847		0.9410	
Predicted R²	0.9036		0.9444		0.8289	
Adequate precision	28.3038		32.3642		19.8035	
Model	Quadratic		Quadratic		Quadratic	

A: Polymer concentration (% *w*/*w*); B: oil concentration (% *v*/*v*); C: S_mix_ concentration (% *v*/*v*); Y_1_: viscosity (cP); Y_2_: in vitro drug release (%); Y_3_: drug content (%); *: significant.

**Table 3:** Characterization of the optimized Ginger extract niosomal gel and emulgel.

Properties	Ginger Niosomal Gel	Ginger Niosomal Emulgel
Visual inspection	Smooth and homogenous	Smooth and homogenous
pH	6.47± 0.31	6.62 ± 0.45
Spreadability (mm)	47.25± 2.4	36.54 ± 3.03 *
Viscosity (cP)	8686.67 ± 210.49	9510.33 ± 162.79 *

Values are stated as mean ± (SD). * *p* < 0.05 compared to Ginger niosomal gel.

**Table 4:** Variables of central composite design for ginger extract emulgel formulations showing independent variables and their level of variation.

Independent Variable	Symbol	Level of Variation	
		−1	+1
Polymer concentration (%*w*/*w*) Oil concentration (%*v*/*v*)	A B	4 10	6 20
S_mix_ concentration (%*v*/*v*)	C	5	10
**Independent Variable**	**Symbol**	**Constrains**	
Viscosity (cP)	Y_1_	In range	
In vitro drug release (%)	Y_2_	Maximize	
Drug content (%)	Y_3_	Maximize	

## Data Availability

Not applicable.

## Supplementary Materials

The following supporting information can be downloaded at: https://www.mdpi.com/article/10.3390/gels8110737/s1, Figure S1: A full UV spectrum graph for the ginger extract.
