# Supplementary material for: Ginger Extract-Loaded Sesame Oil-Based Niosomal Emulgel: Quality by Design to Ameliorate Anti-Inflammatory Activity
Source: Gels. 2022 Nov 14;8(11):737. doi: 10.3390/gels8110737 (PMC9689995; doi:10.3390/gels8110737)
Supplement: Supplementary file 1 [file gels-08-00737-s001.zip › gels-2023376-supplementary.pdf]

gels

Supplementary material

Ginger Extract-Loaded Sesame Oil-Based Niosomal Emulgel: Quality by Design to Ameliorate Anti-Inflammatory Activity

Marwa H. Abdallah [1,2,*], Hanaa A. Elghamry [2], Nasrin E. Khalifa [1,3], Weam M. A. Khojali [4,5], El-Sayed Khafagy [6,7], Amr S. Abu Lila [1,2], Hemat El-Sayed El-Horany [8,9] and Shaimaa El-Housiny [10]

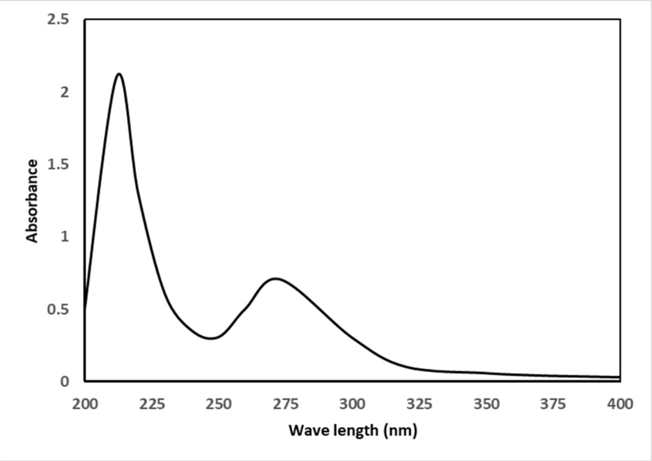

Figure S1. A full UV spectrum graph for the ginger extract.
